# Hypoxia and tissue destruction in pulmonary TB

**DOI:** 10.1136/thoraxjnl-2015-207402

**Published:** 2016-05-31

**Authors:** Moerida Belton, Sara Brilha, Roido Manavaki, Francesco Mauri, Kuldip Nijran, Young T Hong, Neva H Patel, Marcin Dembek, Liku Tezera, Justin Green, Rachel Moores, Franklin Aigbirhio, Adil Al-Nahhas, Tim D Fryer, Paul T Elkington, Jon S Friedland

**Affiliations:** 1 Section of Infectious Diseases and Immunity, Imperial College London, London, UK; 2 Department of Radiology, School of Clinical Medicine, University of Cambridge, Cambridge, UK; 3 Department of Histopathology, Hammersmith Campus, Imperial College London, London, UK; 4 Radiological Science Unit Charing Cross Campus, Department of Nuclear Medicine, Charing Cross Campus, Imperial College NHS Trust, London, UK; 5 Wolfson Brain Imaging Centre, School of Clinical Medicine, University of Cambridge, Cambridge, UK; 6 NIHR Respiratory Biomedical Research Unit, Faculty of Medicine, University of Southampton, Southampton, UK

**Keywords:** Tuberculosis

## Abstract

**Background:**

It is unknown whether lesions in human TB are hypoxic or whether this influences disease pathology. Human TB is characterised by extensive lung destruction driven by host matrix metalloproteinases (MMPs), particularly collagenases such as matrix metalloproteinase-1 (MMP-1).

**Methods:**

We investigated tissue hypoxia in five patients with PET imaging using the tracer [^18^F]-fluoromisonidazole ([^18^F]FMISO) and by immunohistochemistry. We studied the regulation of MMP secretion in primary human cell culture model systems in normoxia, hypoxia, chemical hypoxia and by small interfering RNA (siRNA) inhibition.

**Results:**

[^18^F]FMISO accumulated in regions of TB consolidation and around pulmonary cavities, demonstrating for the first time severe tissue hypoxia in man. Patlak analysis of dynamic PET data showed heterogeneous levels of hypoxia within and between patients. In *Mycobacterium tuberculosis* (*M.tb)*-infected human macrophages, hypoxia (1% pO_2_) upregulated MMP-1 gene expression 170-fold, driving secretion and caseinolytic activity. Dimethyloxalyl glycine (DMOG), a small molecule inhibitor which stabilises the transcription factor hypoxia-inducible factor (HIF)-1α, similarly upregulated MMP-1. Hypoxia did not affect mycobacterial replication. Hypoxia increased MMP-1 expression in primary respiratory epithelial cells via intercellular networks regulated by TB. HIF-1α and NF-κB regulated increased MMP-1 activity in hypoxia. Furthermore, *M.tb* infection drove HIF-1α accumulation even in normoxia. In human TB lung biopsies, epithelioid macrophages and multinucleate giant cells express HIF-1α. HIF-1α blockade, including by targeted siRNA, inhibited TB-driven MMP-1 gene expression and secretion.

**Conclusions:**

Human TB lesions are severely hypoxic and *M.tb* drives HIF-1α accumulation, synergistically increasing collagenase activity which will lead to lung destruction and cavitation.

**Key messages**

**What is the key question?**

- Is there functionally important hypoxia within human pulmonary TB lesions?

**What is the bottom line?**

- Human TB lesions are severely hypoxic, and hypoxia exacerbates matrix metalloproteinase (MMP)-mediated inflammatory tissue destruction.

**Why read on?**

- This study uses PET-CT to demonstrate for the first time that human lung lesions are severely hypoxic and demonstrates that hypoxia, at the level seen in humans, potently upregulates matrix metalloproteinase-1 (MMP-1), a collagenase central to both cavity formation and spread of infection.

## Introduction

*Mycobacterium tuberculosis* (*M.tb*) is solely a pathogen of humans and infects one-third of the global population, killing 1.5 million people each year.^1^ Drug resistance in TB is increasing and there are relatively few new drugs on the horizon. Novel approaches to therapy require an understanding of the immunopathology of TB. Lung tissue destruction is a hallmark of pulmonary TB^2^ and is key for transmission of infection. Reduced tissue oxygenation has been noted in animal models of TB,^3^ ^4^ but hypoxia in human disease has not been investigated although it may have a significant effect on the host response. The pathology of human TB is different from most animal models.^2^ Furthermore, although extensive lung damage is found in TB, the effect of hypoxia on proinflammatory tissue destruction in TB has not been explored.

Matrix metalloproteinases (MMPs) are emerging as key proteases causing TB immunopathology. Recent evidence implicates MMPs, particularly collagenases such as matrix metalloproteinase-1 (MMP-1), as key in driving tissue destruction during pulmonary TB.^5–7^ Since MMPs may cause uncontrolled proteolytic destruction, MMP activity is tightly regulated at the transcriptional level, by cleavage of the proenzyme to an active form and by specific tissue inhibitors of metalloproteinases (TIMPs) which negatively regulate protease activity. Regulation of MMPs by hypoxia in TB has not been studied.

The cellular response to hypoxia is orchestrated by hypoxia-inducible factor (HIF)-1, a heterodimeric transcription factor considered a master regulator of the host response to oxygen deprivation.^8^ ^9^ In addition to regulating oxygen homeostasis, emerging evidence implicates HIF-1 in infectious and inflammatory diseases.^10^ HIF-1 is comprised of two subunits: oxygen-responsive HIF-1α and constitutively expressed HIF-1β. In normoxia, HIF-1α is hydroxylated by prolyl-4-hydroxylase proteins (PHDs),^11^ ^12^ allowing binding of the von Hipple–Lindau protein which targets HIF-1α for proteosomal degradation.^13^ Under hypoxic conditions, PHD activity declines, resulting in HIF-1α accumulation and HIF-1 heterodimer stabilisation.^14^ This HIF-1 complex binds to promoter regions of hypoxia-inducible genes, upregulating an array of genes, including those involved in cell survival, angiogenesis, apoptosis, erythropoiesis, glucose metabolism and pH regulation.^11^ HIF is responsive to a variety of stimuli, with the inflammatory transcription factor NF-κB being of particular importance in modulation of HIF expression.^9^ The complex interaction between HIF-1α and NF-κB signifies a synergistic link between hypoxia and immune responses, indicating a potential role of hypoxia in driving TB immunopathology.

In this study, we show for the first time that lesions in pulmonary TB infection are severely hypoxic in man. There is heterogeneity both within and between lesions in the extent of hypoxia. In primary human monocyte-derived macrophages (MDMs) and normal human bronchial epithelial cells (NHBEs), hypoxia synergistically upregulates HIF-1α-dependent MMP-1 (collagenase) gene expression and secretion during *M.tb* infection. In addition, *M.tb* stabilises HIF-1α even in the absence of hypoxia. HIF-1α accumulation is necessary for MMP-1 secretion and HIF-1α is highly expressed in macrophages in human TB granulomas.

## Methods

Full methods are provided in the online supplement.

10.1136/thoraxjnl-2015-207402.supp1 Supplementary data

### Patient recruitment

Patients were recruited from Imperial College Healthcare NHS Trust with a confirmed microbiological diagnosis of TB, either on acid-fast smear and/or *M.tb* culture. All patients had abnormal plain chest radiographs and had received less than 2 weeks of anti-TB therapy. Informed consent was obtained from all study participants.

### ^18^F-MISO PET-CT scans

PET-CT scans were performed on a Siemens mCT (Siemens Medical, Erlangen, Germany) at the Department of Nuclear Medicine Charing Cross Hospital, Imperial College NHS Trust, London. [^18^F]fluoromisonidazole ([^18^F]FMISO) was synthesised in the Wolfson Brain Imaging Centre, University of Cambridge. Patient scanning protocol and data analysis are described in the online supplement.

### *M.tb* culture

*M.tb* H37Rv was cultured in Middlebrook 7H9 medium as previously described.^15^

### Cell culture experiments

Monocyte-derived primary human macrophages were infected with *M.tb* H37Rv as described.^15^ Primary NHBEs (Lonza, Slough, UK) were cultured and stimulated with conditioned media from *Mtb*-infected monocytes (CoMTb) and A549 cells transiently transfected as described.^16^

### Hypoxia workstation

A custom-designed hypoxia workstation was commissioned for the biological safety level 3 facility (Coy Laboratories, USA).

### Casein zymography

Casein zymography was performed as previously described.^15^

### Real-time PCR

Macrophages were lysed using Tri-Reagent (Sigma-Aldrich, Dorset, UK), and total RNA was extracted using PureLink RNA Mini Kit (Invitrogen, Paisley, UK). RNA was reverse transcribed using QuantiTect Reverse Transcriptase Kit (Qiagen, Manchester, UK). Quantitative PCR reactions were performed in an ABI Prism 7700 (Applied Biosystems, Paisley, UK).

#### Measurement of MMP and TIMP concentrations

Total MMP and TIMP secretion in cell culture supernatants was measured by ELISA (R&D Systems, Abdingdon, UK) or on the Luminex200 platform using MMP Luminex multiplex array (R&D Systems) according to the manufacturer's instructions. The minimum level of detection for MMP-1 was 10 pg/mL.

#### HIF-1α western analysis

Western blotting was performed using anti-HIF1α Ab (BD Biosciences, UK) and goat anti-mouse IgG horseradish peroxidase (HRP)-conjugate secondary Ab (Jackson ImmunoResearch). Loading control was performed using the rabbit-anti-β-actin Ab and goat anti-mouse IgG HRP-conjugate secondary Ab (Jackson ImmunoResearch). Luminescence was detected with the enhanced chemiluminescence (ECL) system (Amersham, UK) according to the manufacturer's protocol.

#### Confocal microscopy

Primary human MDMs were infected with *M.tb* H37RV in Permanox plastic chamber slides (Thermo Fisher Scientific, UK). MMP-1 staining was performed using anti-MMP-1 primary Ab (Abcam, UK) and goat anti-mouse secondary Ab (Abcam) according to the manufacturers' instructions. Nuclei were visualised using 4',6-diamidino-2-phenylindole (DAPI).

#### siRNA

MDMs were transfected using DharmaFECT 3 transfection reagent with either non-targeting (NT) control small interfering RNA (siRNA) or HIF-1α siRNA smartpool (Dharmacon, Fisher Scientific, Loughborough, UK), according to the supplier's instructions.

#### Immunohistochemistry

HIF-1α immunohistochemistry was performed on formalin-fixed, paraffin-embedded lung biopsies from six patients with culture-proved *M.tb* infection and six non-infected control samples. Immunohistochemistry staining was performed using Bond III fully automated staining system with the Bond Polymer Refine Detection system and associated reagents (Leica Microsystems, Newcastle-Upon-Tyne, UK). HIF-1α was detected using HIF1-α antibody (Abcam AB1).

#### Statistics

Statistical analysis was performed using GraphPad PRISM 6. Data were analysed with Kruskal–Wallis for comparison between three or more groups and Dunn's test for pairwise comparisons. A p value of 0.05 was considered significant. For all experiments, bars represent mean values±SD from a minimum of two independent experiments performed in triplicate.

## Results

### Regions of severe hypoxia are present in human pulmonary TB lesions

**Figure 1:**
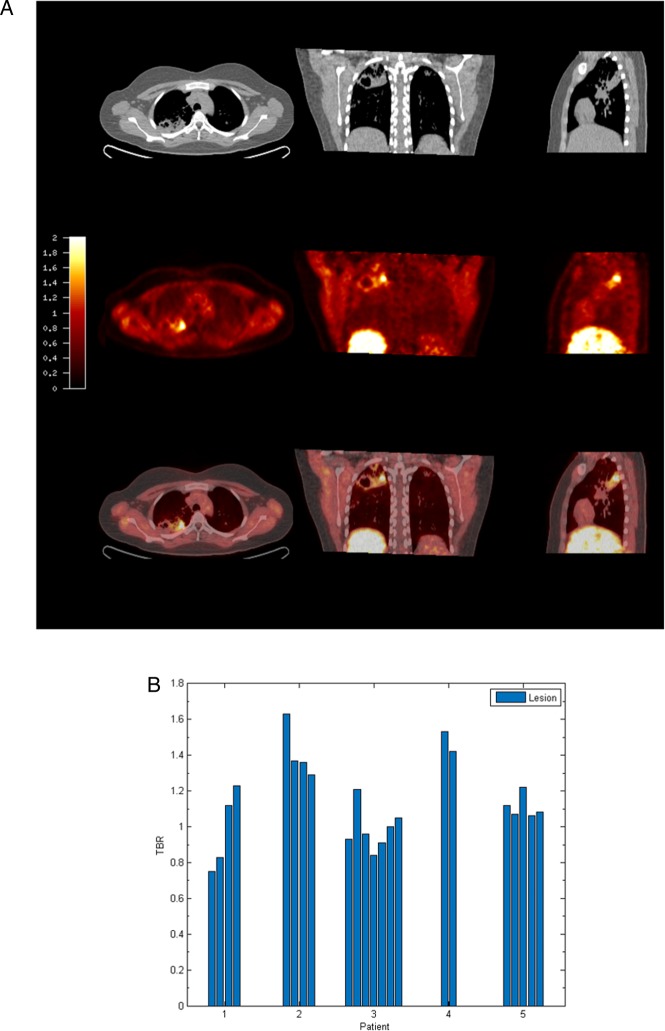
[^18^F] fluoromisonidazole ([^18^F]FMISO) PET-CT demonstrates increased tracer uptake within TB lesions. (A) [^18^F]FMISO PET-CT images. Transverse, coronal and sagittal slices through the [^18^F]FMISO target-to-background (TBR) and CT images of Patient 2. Top row, CT images show consolidation and a pulmonary cavity in the right upper lobe with a smaller, non-cavitating lesion in the left lung. Middle row, [^18^F]FMISO TBR map demonstrates intense uptake of tracer in the right upper lobe lesion and the liver, the site of metabolism of [^18^F]FMISO. Bottom row, coregistered CT and TBR images. (B) TBR values greater than unity were found for at least one lesion in all patients (each individual bar represents one region of interest, ROI).

To investigate hypoxia in patients with TB, we performed PET-CT scans in patients who had acid-fast bacilli identified in respiratory specimens that were subsequently culture confirmed to be *M.tb* (patient demographics and microbiology are in online supplementary table S1)*.* The average duration of pulmonary symptoms was 2.4 months and all patients were HIV negative (patient laboratory data are presented in online supplementary table S2). To investigate hypoxia, we used the hypoxia-specific tracer [^18^F]FMISO, which has been used to study tumour biology.^17^ [^18^F]FMISO is selectively trapped in severely hypoxic regions (pO_2_<10 mm Hg).^18–20^ PET-CT scans in five patients demonstrated extensive uptake of [^18^F]FMISO in radiologically abnormal areas of the lung, as assessed by [^18^F]FMISO target-to-background (TBR) (figure 1A), with the lateral muscle used as a background representing normoxic tissue. TBR values significantly greater than unity were found in one or more lesions in all patients (figure 1B).

**Figure 2:**
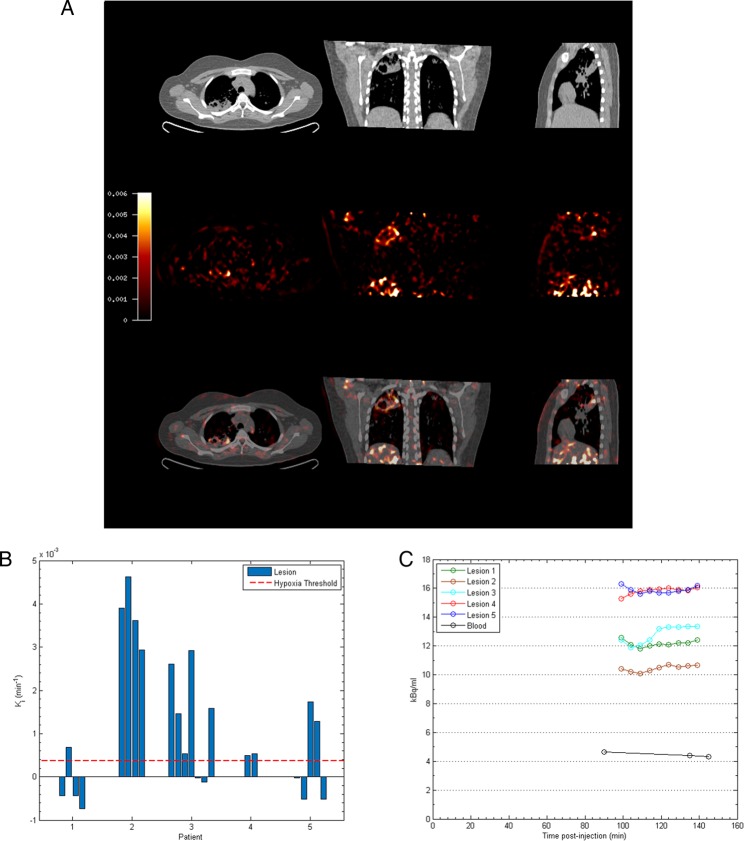
Severe hypoxia is present within human pulmonary TB lesions. (A) Patlak K_i_ images from dynamic PET-CT scanning for Patient 2 demonstrate intense retention of [^18^F] fluoromisonidazole ([^18^F]FMISO) in the right upper lobe of the lung (middle row). Coregistered PET-CT images (bottom row) confirm localisation of hypoxia to the region of the right upper lobe cavity. (B) Regional K_i_ values demonstrate heterogeneous trapping of [^18^F]FMISO within and between patients. The hypoxia threshold (0.00037/min) was determined from the mean+3 SDs of the Ki values in the normoxic lateral muscle background regions across the patient group. (C) Time–activity measurements of five regions of interest (ROIs) from Patient 3 demonstrate high tissue levels compared with peripheral blood values.

To provide a more specific measure of hypoxia than TBR, Patlak K_i_ mapping was applied, which demonstrated the presence of severe hypoxia within areas of consolidation and in the regions immediately surrounding pulmonary cavities (see figure 2A and online supplementary table S3). All patients had K_i_ values greater than the hypoxia threshold in at least one region of interest (ROI) (figure 2B). Heterogeneous levels of hypoxia were seen within patients with the exception of Patient 2, who demonstrated evidence of severe hypoxia in all ROIs. Finally, time–activity curves demonstrated significantly higher levels of [^18^F]FMISO within ROIs compared with peripheral blood (figure 2C). Together, these data demonstrate for the first time the presence of severe hypoxia within TB lesions in man.

### Hypoxia increases *M.tb*-driven MMP-1 expression in human macrophages

**Figure 3:**
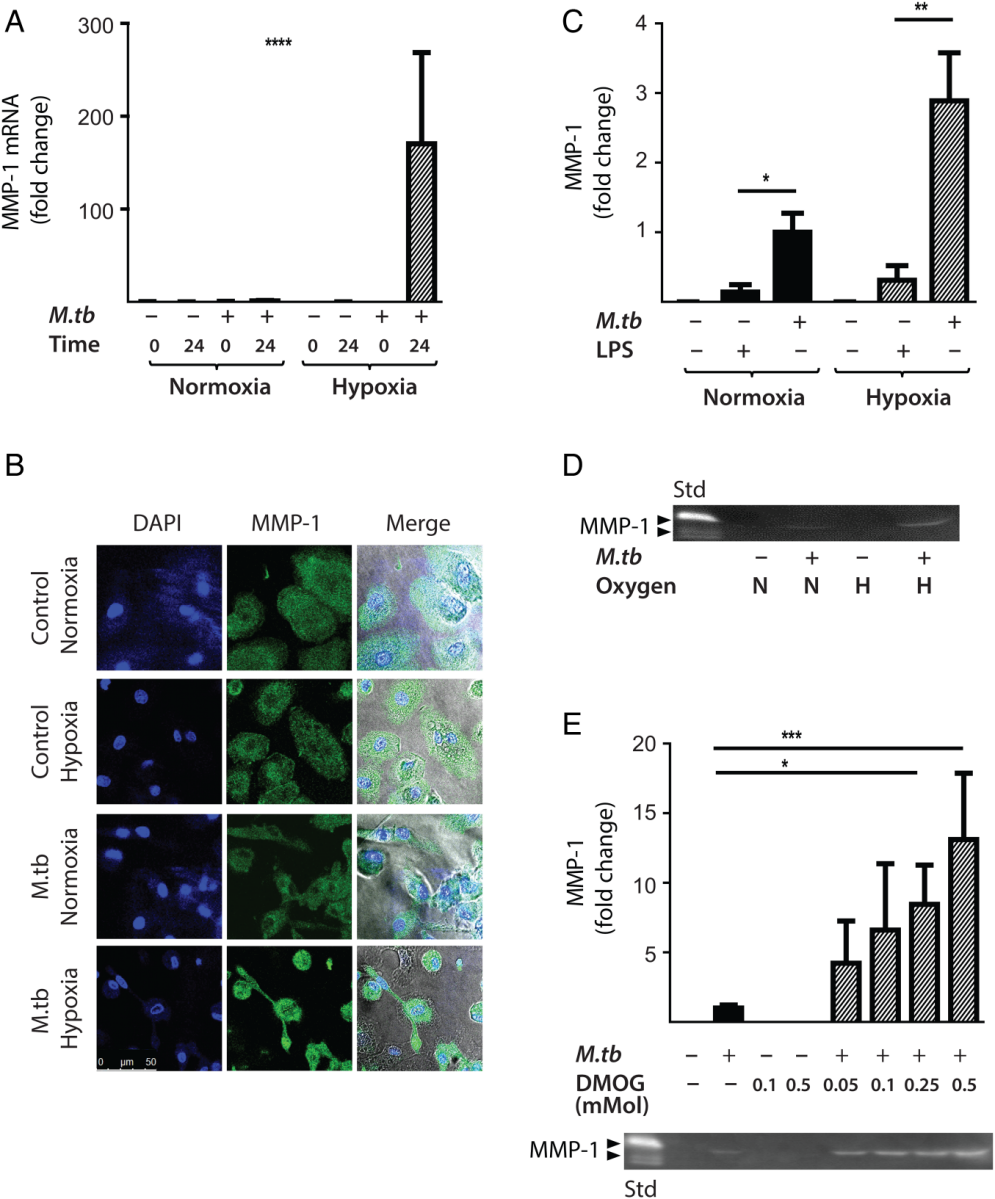
Hypoxia increases *Mycobacterium tuberculosis (M.tb)*-driven matrix metalloproteinase-1 (MMP-1) expression and secretion by human monocyte-derived macrophages (MDMs). (A) Hypoxia (1% pO_2_) increases MMP-1 gene expression in primary human MDMs infected with *M.tb* at 24 h. (B) Hypoxia increases intracellular MMP-1 accumulation on confocal microscopy at 72 h in MDMs infected with *M.tb* (MOI=1) compared with normoxia or control uninfected cells. (C) Mtb infection increases MMP-1 secretion by infected MDMs greater than LPS (100 ng/mL) in both normoxia and hypoxia analysed 72 h after infection. (D) MMP-1 activity is increased in *M.tb*-infected but not control human macrophages analysed by casein zymography, and hypoxia further increases caseinolytic activity. (E) Stabilisation of hypoxia-inducible factor (HIF)-1α by dimethyloxalyl glycine (DMOG) (range 0.05–0.5 mM) significantly increases MMP-1 secretion analysed by ELISA and proteolytic activity measured by zymography in a dose-dependent manner. N=21% O_2_; 5% CO_2_, H=1% O_2_; 5% CO_2_. ****p<0.0001, ***p<0.001, **p<0.01, *p<0.05. LPS, lipopolysaccharide.

Next, the effect of hypoxia (1% pO_2_) on gene expression and secretion of MMP-1 was investigated. Hypoxia significantly increased MMP-1 gene expression in *M.tb*-infected human MDMs compared with infection in normoxia (21% pO_2_), resulting in a 170-fold increase in MMP-1 mRNA accumulation at 24 h (figure 3A, p<0.0001 by Kruskal–Wallis test). Increased MMP-1 accumulation under hypoxic conditions in *M.tb*-infected MDMs was confirmed by confocal microscopy (figure 3B). In both normoxia and hypoxia, *M.tb* was a more potent stimulus to MMP-1 secretion than lipopolysaccharide (LPS) (figure 3C, p<0.05 and p<0.01, respectively). Casein zymography demonstrated that the increased MMP-1 secretion in hypoxia was proteolytically active (figure 3D).

Next, we used dimethyloxalyl glycine (DMOG) to block prolyl hydroxylase activity, thereby stabilising HIF-1α and activating the HIF-1 pathway. Consistent with findings in 1% pO_2_, DMOG significantly increased MMP-1 secretion in *M.tb*-infected macrophages, causing a dose-dependent increase in secretion and caseinolytic activity (figure 3E). Hypoxia caused no difference in *M.tb* CFU 72 h post-infection (data not shown), demonstrating that intracellular replication of *M.tb* was not altered.

### Hypoxia increases *M.tb*-dependent MMP-1 expression in respiratory epithelial cells

**Figure 4:**
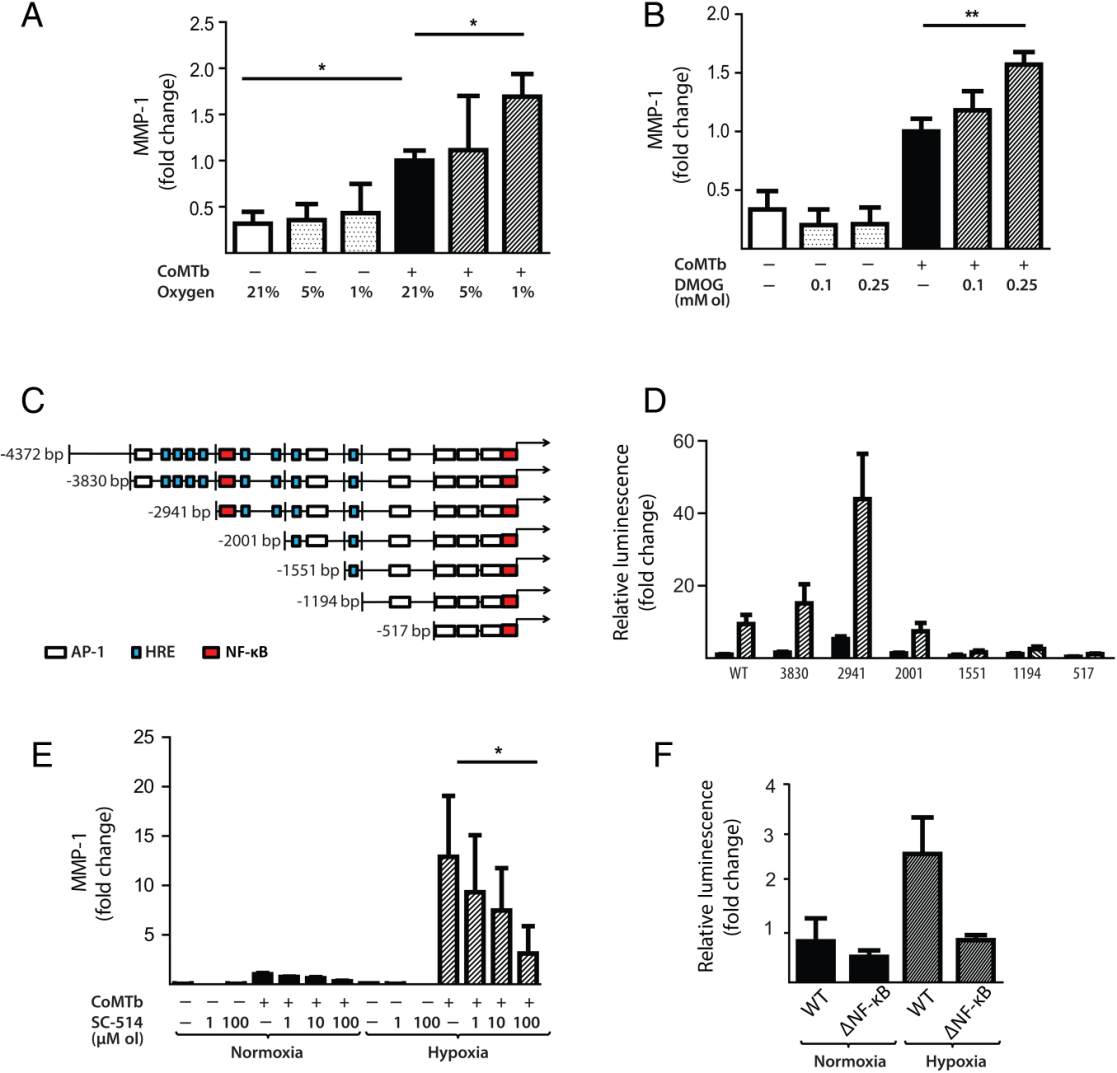
Hypoxia upregulates matrix metalloproteinase-1 (MMP-1) in human respiratory epithelial cells. (A) Hypoxia increases MMP-1 secretion from normal human bronchial epithelial (NHBE) cells stimulated with control medium or conditioned media from *Mtb*-infected monocytes (CoMTb) incubated in 21%, 5% or 1% oxygen. (B) Dimethyloxalyl glycine (DMOG) (range 0.1–0.25 mM) drives dose-dependent MMP-1 secretion from CoMTb-stimulated NHBE cells. (C) In silico analysis of the MMP-1 promoter reveals putative hypoxic response element (HRE)-binding sites as well as consensus NF-κB-binding and AP-1-binding sites. (D) Relative luminescence following transfection of A549 respiratory epithelial cells with either WT-MMP-1 promoter or a series of MMP-1 promoter deletion constructs in normoxia (solid bars) or hypoxia (shaded bars). The effect of hypoxia in increasing promoter activity is absent in constructs −1551, −1194 and −517. (E) IKK-β inhibition in hypoxia with SC-514 in CoMTb-stimulated cells resulted in a dose-dependent decrease in MMP-1 secretion. (F) Site-directed mutagenesis of the NF-κB-binding site at −2878 to −2886 bp decreases MMP-1 promoter activity in response to CoMTb in normoxia (solid bars) or hypoxia (shaded bars). **p<0.01, *p<0.05. AP-1, activated protein-1; IKK-β, inhibitor of nuclear factor kappa-B kinase subunit beta; WT, wild type.

Next, we investigated the effect of hypoxia on respiratory epithelial cell MMP-1 secretion, as stromal cell networks are important sources of MMPs in TB.^16^ CoMTb stimulation of primary NHBEs in hypoxia (5% and 1% pO_2_) increased MMP-1 secretion (figure 4A; p<0.05). Lactate dehydrogenase (LDH) release assay showed that hypoxia did not increase cell cytotoxicity (see online supplementary figure S1). Such MMP-1 secretion is potentially open to modification by small molecules, since stabilisation of HIF-1α by DMOG also increased MMP-1 secretion in CoMTb-stimulated NHBEs in a dose-dependent manner (figure 4B, p<0.01). In addition, 0.25 mM DMOG increased NHBE cell MMP-9 secretion and proteolytic activity on gelatin zymography (see online supplementary figure S2).

There were similar findings in the A549 respiratory epithelial cell line (see online supplementary figure S3). In silico analysis of the MMP-1 promoter sequence demonstrated numerous putative hypoxia response elements which are HIF-1-binding sites, as well as known NF-κB and activated protein-1 (AP-1)-binding sites (figure 4C). A549 cells were transiently transfected with either full-length MMP-1 promoter-reporter genes or truncation constructs and stimulated with CoMTb in the presence or absence of hypoxia. In normoxia, deletions upstream of −2941 resulted in increased MMP-1 promoter activity indicating the presence of inhibitory elements (figure 4D). Deletions downstream of −2941 bp from the transcriptional start site reduced MMP-1 promoter activity. The presence of hypoxia resulted in increased MMP-1 promoter activity, compared with normoxia, in deletion constructs upstream of −1551. To investigate whether NF-κB regulated MMP-1 expression in hypoxia, inhibitor of nuclear factor kappa-B kinase subunit beta (IKK-β) activity was inhibited by SC-514. IKK-β inhibition significantly decreased MMP-1 secretion in a dose-dependent manner in normoxia and this effect was more pronounced in hypoxia (figure 4E). Site-directed mutagenesis of the NF-κB-binding site between −2878 and −2886 bp in the MMP-1 promoter reduced MMP-1 promoter activity observed in hypoxia (figure 4F; p<0.01 by Kruskal–Wallis).

### *M.tb* stabilises HIF-1α, driving MMP-1 expression, and HIF-1α is expressed in TB patient granulomas

**Figure 5:**
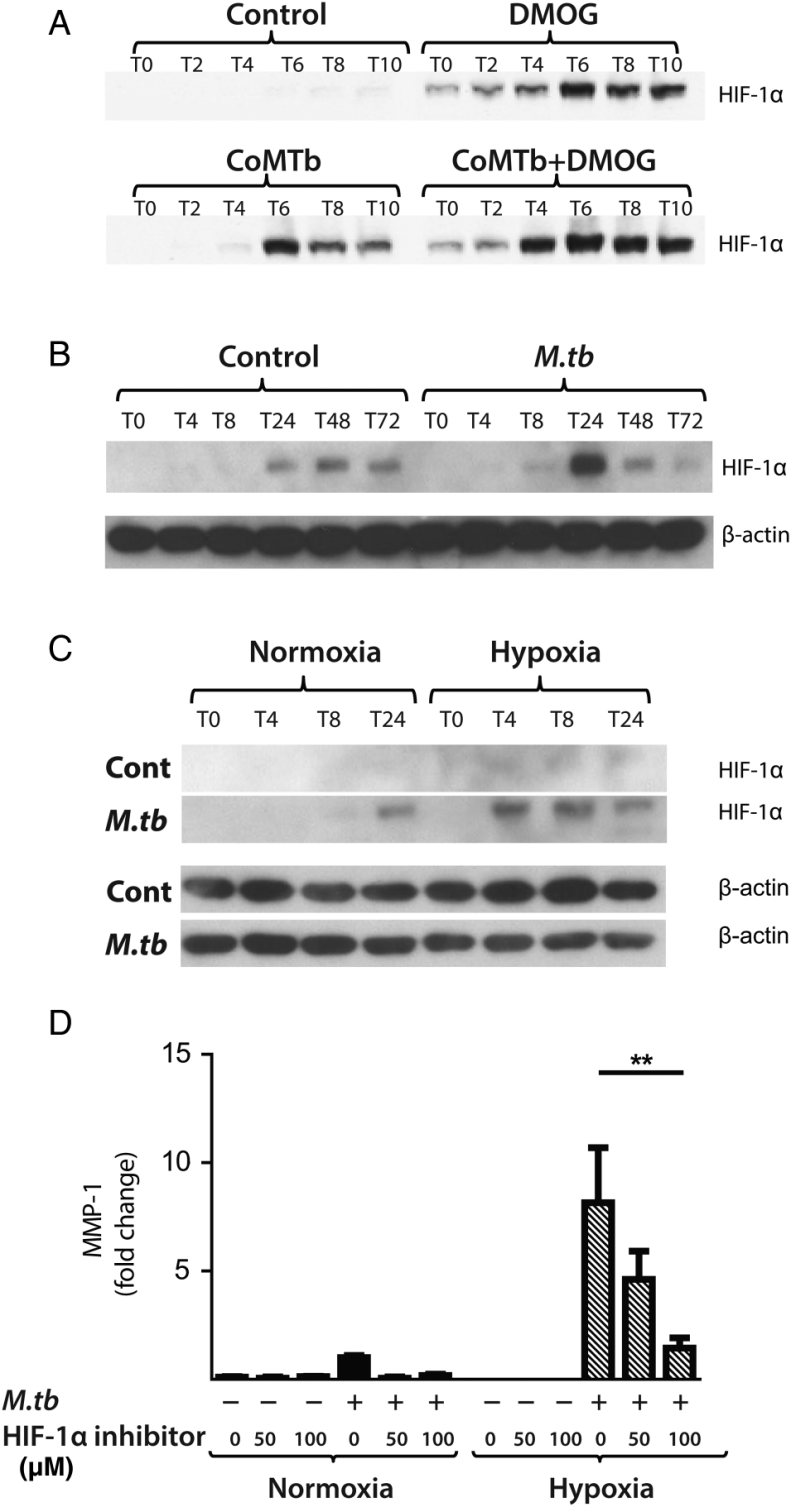
*Mycobacterium tuberculosis (M.tb)* infection drives hypoxia-inducible factor (HIF)-1α accumulation in normoxia. (A) In A549 cells, conditioned media from *Mtb*-infected monocytes (CoMTb) stimulation and dimethyloxalyl glycine (DMOG) (0.25 mM) result in early HIF-1α stabilisation. Preincubating A549 cells with DMOG (0.25 mM) and subsequent stimulation with CoMTb markedly increase HIF-1α accumulation, peaking at 6 h. (B) *M.tb* infection increases HIF-1α accumulation and stabilisation in normoxia in primary human monocyte-derived macrophages (MDMs), peaking at 24 h. (C) Combined infection with *M.tb* and exposure to hypoxia cause greater HIF-1α accumulation than either stimulus alone, peaking at 4 h and persisting until 24 h. (D) Inhibition of HIF-1α by LW6 (range 50–100 µM) results in decreased matrix metalloproteinase-1 (MMP-1) secretion in *M.tb*-infected MDMs in hypoxia (hatched bars). **p<0.01.

Next, we investigated whether *M.tb* directly affected HIF-1α accumulation independently of the stimulus from hypoxia. In respiratory epithelial cells, DMOG and CoMTb stimulation of A549 cells independently upregulated HIF-1α. Concurrent exposure to DMOG and stimulation by CoMTb resulted in maximal accumulation of HIF-1α (figure 5A). In human MDMs, *M.tb* infection in normoxia induced stabilisation and accumulation of HIF-1α peaking at 24 h (figure 5B). Hypoxia and *M.tb* infection synergistically drove prolonged and increased HIF-1α accumulation in MDMs compared with cells exposed to either stimulus alone, with HIF-1α accumulation detectable from 4 h after infection (figure 5C). *M.tb* infection did not induce HIF-2 accumulation either in normoxia or hypoxia (data not shown).

**Figure 6:**
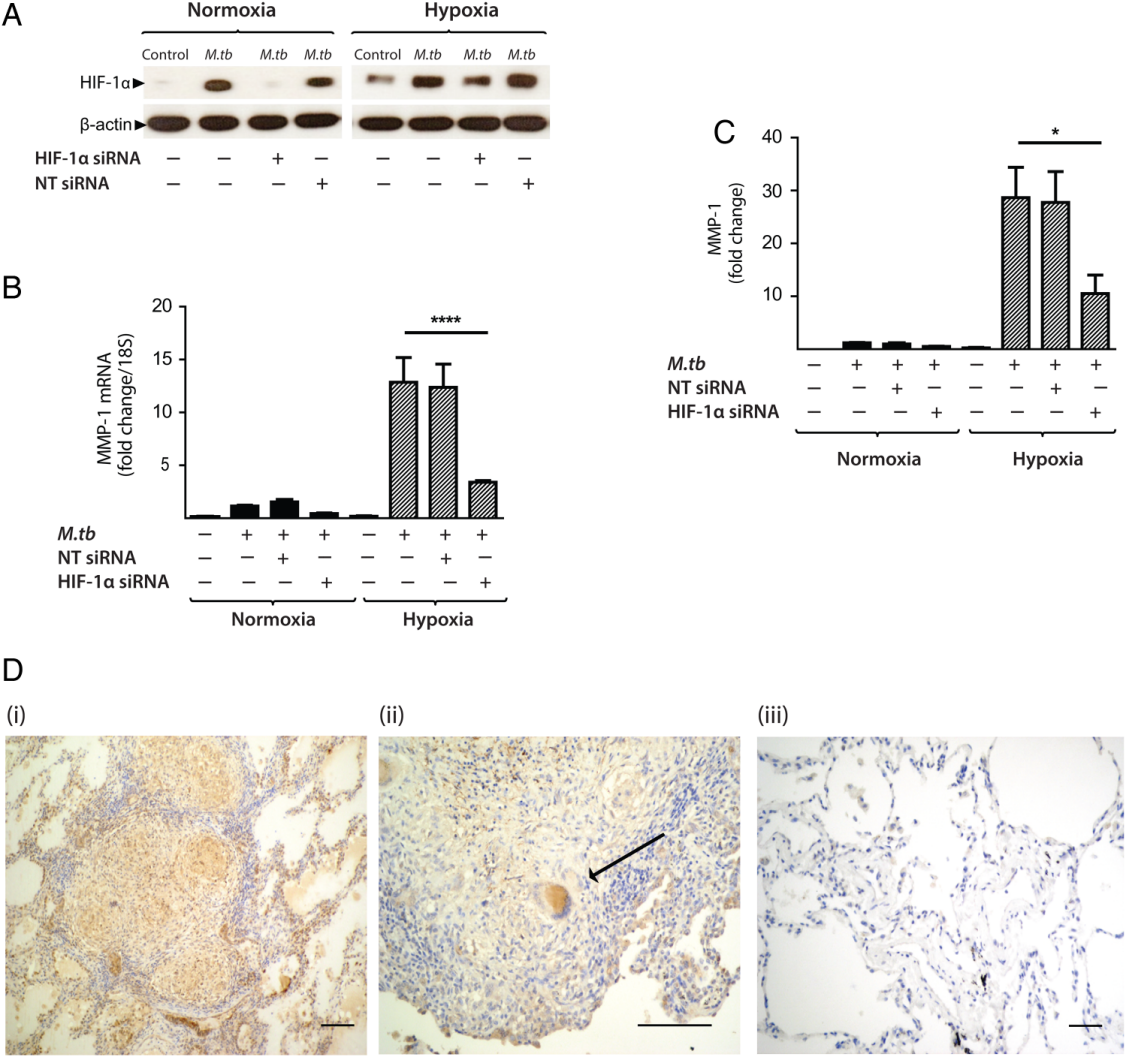
Hypoxia-inducible factor (HIF)-1α is necessary for matrix metalloproteinase-1 (MMP-1) gene expression and secretion during *Mycobacterium tuberculosis (M.tb)* infection. (A) HIF-1α western blot at 24 h demonstrates suppression of HIF-1α protein by HIF-1α siRNA in monocyte-derived macrophages (MDMs) in both normoxia and hypoxia, with no knockdown observed with non-targeting (NT) siRNA. (B) HIF-1α siRNA decreases MMP-1 gene expression at 24 h in *M.tb*-infected MDMs incubated in hypoxia. (C) HIF-1α siRNA causes a significant decrease in MMP-1 secretion at 48 h under conditions of normoxia (solid bars) and hypoxia (hatched bars). No significant change in MMP-1 gene expression or secretion is seen with NT siRNA. (D) Lung biopsies from patients with pulmonary TB (i, ii) are immunoreactive for HIF-1α compared with uninfected control biopsies (iii). HIF-1α staining is pronounced within macrophages and multinucleated giant cells (arrow, ii). Scale bars, 100 µm. ****p<0.0001, *p<0.05. siRNA, small interfering RNA.

To investigate whether HIF-1α regulated MMP-1 expression during TB infection, we inhibited the activity of HIF-1α in normoxia and hypoxia. The inhibitor LW6 caused a trend to decreased *M.tb*-driven MMP-1 secretion in human MDMs in normoxia, but in hypoxia there was a significant dose-dependent decrease in MMP-1 secretion (figure 5D; p<0.05). To confirm this observation, further studies were performed with HIF-1α siRNA. Western blot analysis demonstrated that targeted but not NT siRNA decreased HIF-1α protein in *M.tb*-infected MDMs in normoxia and hypoxia (figure 6A). HIF-1α siRNA decreased MMP-1 mRNA accumulation (figure 6B; p<0.0001) and protein secretion (figure 6C) in both normoxic and hypoxic conditions (p<0.05). NT siRNA did not suppress MMP-1 gene expression or protein secretion.

To investigate the relevance of HIF-1α to TB granulomas in patients, we performed immunohistochemical analysis of lung biopsy specimens from patients with a confirmed diagnosis of TB. Epithelioid macrophages and Langerhans giant cells were immunoreactive for HIF-1α staining within TB granulomas (figure 6D i,ii) compared with uninfected control lung tissue (figure 6D iii), demonstrating that TB infection causes HIF-1α accumulation.

## Discussion

In this first detailed study of hypoxia in human TB, we demonstrate the presence of severe hypoxia within areas of lung consolidation and around pulmonary cavities in human TB. Hypoxia increases gene expression and secretion of MMP-1, a key collagenase that causes tissue destruction and immunopathology in pulmonary TB. In addition, *M.tb* infection directly drives HIF-1α accumulation, which is further increased in hypoxia to synergistically activate immunopathogenic signalling pathways.

We demonstrated hypoxia in human TB using [^18^F]FMISO PET-CT scanning for the first time in patients with an infectious disease. Hypoxia has not previously been found in man although reported in animal models of TB, all of which have somewhat different pathology to human disease.^3^ ^4^ Until now, the investigation of hypoxia and TB has focused primarily on pathogen responses to oxygen restriction, controlled by the two-component regulation system dosR/dosS (or dosR regulon).^21^ ^22^ Direct assessment of hypoxia *in vivo* typically involves the utilisation of polarographic oxygen electrodes which can provide absolute measurements of tissue oxygenation at the sampling location. However, this procedure is technically demanding and suffers from a number of limitations, including invasiveness, high susceptibility to sampling errors and the fact that only easily accessible locations can be interrogated.^23^ [^18^F]FMISO PET-CT scans define regions of hypoxia without the need for invasive probes, as previously shown in tumour biology^24^ and cerebral ischaemia.^25^ Since high tracer uptake, as quantified by standardised uptake value or TBR, may represent high tracer delivery to a ROI rather than tracer trapping under hypoxic conditions, we determined the influx rate of [^18^F]FMISO into the trapped tissue compartment (K_i_) so as to more specifically identify tissue hypoxia. The maximum TB lesion K_i_ value (0.005/min) is equal to the highest K_i_ value in an [^18^F]FMISO study of head and neck cancers by Wang *et al*,^26^ which puts the degree of hypoxia in this study into context. Our data reveal that heterogeneity exists both within individual TB lesions and between lesions, consistent with the concept of multiple TB microenvironments existing within a single patient.

The finding of hypoxia within lung lesions directed our cellular experiments. MMP-1 gene expression and secretion were significantly increased in *M.tb*-infected human macrophages in hypoxia compared with normoxia and MMP-1 was functionally active. However, hypoxia had no effect on mycobacterial growth in our experiments. Diverse lines of investigation are implicating MMP-1 as a key protease in TB pathology. MMP-1 causes collagen destruction in *M.tb*-infected transgenic mice^6^ and *M.tb* infection upregulates MMP-1 more potently than *M.bovis* BCG in human macrophages.^15^ In presensitised rabbits, MMP-1 has been shown to have a causal role in pulmonary cavitation.^27^ Specifically, the development of cavities within areas of dense consolidation has been associated with MMP-1/TIMP imbalance and high intracavitary bacterial burden. In human TB granulomas, MMP-1 expression is upregulated 606-fold compared with uninfected lung.^28^ Similarly, MMP-1 was the most potently upregulated gene in macrophages from patients who developed TB compared with those with latent disease.^29^ The effect of hypoxia was mimicked by chemically targeting the pathway with DMOG, which suggests that it may be possible to therapeutically manipulate the excess inflammatory response in TB using small molecules.

Hypoxia-dependent, monocyte-driven upregulation of MMP-1 gene expression in human respiratory epithelial cells required the transcription factor NF-κB. NF-κB and HIF-1α interact to regulate innate immunity and inflammation.^9^ ^10^ NF-κB critically regulates MMP activity in TB infection of the central nervous system,^30^ but has not been investigated in the context of hypoxia-driven MMP expression in TB infection. NF-κB is both directly affected by hypoxia and modulates HIF-1α expression as well as being regulated by prolyl hydroxylases.^31^ The precise nature of the interaction between HIF-1α, prolyl hydroxylases and NF-κB in TB is currently being investigated by our group.

*M.tb* caused significant accumulation of HIF-1α in direct infection and via a networking effect even in the absence of hypoxia, with a synergistic increase in HIF-1α expression in hypoxia or following treatment with DMOG. To our knowledge, this is the first time that hypoxia-independent HIF-1α stabilisation has been observed in any mycobacterial infection, although similar effects have been reported in other infections.^32^ ^33^ Interestingly, recent gene expression profiling in peripheral whole blood demonstrated significant upregulation of HIF-1α gene expression in patients with TB compared with controls.^34^ Our findings are consistent with the demonstration of activation of HIF-1α signalling pathways following infection in a zebrafish model of TB infection.^35^ We showed expression of HIF-1α within TB granulomas, localising the activity to epithelioid macrophages and multinucleate giant cells, which we have previously shown are key in secretion of several MMPs.^15^ We demonstrate that HIF-1α regulates MMP-1 expression in TB. Since the chemical inhibitor LW6 can lack specificity, we used targeted siRNA to confirm this finding. Consistent with our data in TB, hypoxia and HIF-1α regulate MMP-driven tissue destruction in hypoxic fibroblasts within rheumatoid synovium.^36^

In summary, we show for the first time that human TB lesions are severely hypoxic. Hypoxia increases MMP-1 gene expression and secretion during *M.tb* infection, and *M.tb* infection independently increases HIF-1α activity even under conditions of normoxia. Taken together, our data suggest the *M.tb* drives a HIF-1α-dependent proinflammatory tissue destructive cascade that may lead to cavitation and promote disease transmission, representing a potential future therapeutic target in the era of increasing drug-resistant TB.
