# Supplementary material for: Hypoxia and tissue destruction in pulmonary TB
Source: Thorax. 2016 May 31;71(12):1145–53. doi: 10.1136/thoraxjnl-2015-207402 (PMC5136721; doi:10.1136/thoraxjnl-2015-207402)
Supplement: Supplementary data [file thoraxjnl-2015-207402supp.pdf]

# Hypoxia and HIF-1α-dependent MMP-1 secretion in human pulmonary tuberculosis

Moerida Belton, Sara Brilha, Roido Manavaki , Francesco Mauri, Kuldip Nijran, Young T Hong, Neva Patel, Marcin Dembek, Liku Tezera, Justin Green, Rachel Moores, Franklin Aigbirhio, Adil Al-Nahhas, Tim D Fryer, Paul T Elkington, Jon S Friedland

Online data supplement

## Methods

### *$^{18}$F-MISO PET-CT scans*

PET-CT scans were performed on a Siemens mCT (Siemens Medical, Erlangen, Germany) at the Department of Nuclear Medicine Charing Cross Hospital, Imperial College NHS Trust, London. [$^{18}$F]FMISO was synthesized in the Wolfson Brain Imaging Centre, Cambridge University. Patients received 406 ±6 MBq [$^{18}$F]FMISO intravenously followed by a 90 minute uptake period to promote contrast between normoxic and hypoxic tissue on tissue-to-background (TBR) images, and also to allow the free [$^{18}$F]FMISO concentration in tissue to reach equilibrium with that in blood, a pre-requisite for Patlak plot determination of influx rate ($K_i$) [1]. Immediately following a low dose CT scan for attenuation correction, patients underwent a 45 minute single bed position PET scan acquired in list mode. As Patlak analysis requires knowledge of the input function to the tissue, three venous blood samples (90, 135 and 145 minutes post-injection) were taken in order to scale to a population-based [$^{18}$F]FMISO arterial plasma function. The latter was produced from 6 controls scanned at The Wolfson Brain Imaging Centre, University of Cambridge as part of a study on stroke (PI: Prof J-C Baron), which included late venous sampling as part of the study protocol.

### *[$^{18}$F]FMISO PET-CT data analysis*

List mode PET data were reconstructed into 9x5min frames using OSEM [2] (8 subsets, 4 iterations, 5mm FWHM 3D Gaussian post-smoothing filter) into a 168x168x74 array with voxel dimensions 4.07 x 4.07 x 3.00mm. Corrections were applied for randoms, dead time, normalization, scatter, attenuation, sensitivity and radioactive decay. To reduce the impact

of patient motion during PET acquisition, the last 8 frames of the dynamic PET image series were non-rigidly registered to the first frame using symmetric normalization (SyN) with mutual information as the similarity metric [3]. All PET frames were then registered to CT using affine transformation. The last three of the registered frames (120-135mins post injection) were converted to a TBR image through normalization with the radioactivity concentration in a bilateral lateral muscle region assumed to represent normoxic tissue. All 9 frames were used to produce a $K_i$ map using Patlak plot analysis. Regions of interest (ROI) were defined on the TBR images and ROI values were determined by placing the ROIs on the TBR and $K_i$ maps.

### *M.tb* culture

*M.tuberculosis* H375v was cultured in Middlebrook 7H9 medium supplemented with 10% OADC enrichment medium (BD Biosciences, Oxford, UK), 0.2% glycerol and 0.02% Tween 80. Cultured cells were infected with *M.tb* at mid-logarithmic growth at an optical density of 0.6 (Biowave cell density meter, WPA, Cambridge, UK). To count intracellular bacilli, infected MDMs were lysed with 0.1% Triton-X. Aliquots of cell lysates were serially diluted 10-fold in 7H9 liquid and 4 dilutions of each sample were plated on 7H11 agar and incubated for 14 days befoe determining the number of CFU.

### ***Monocyte Purification and Maturation***

Monocytes were isolated from single donor leucocyte cones (National Blood Transfusion Service, London, UK) by density gradient centrifugation (Amersham Biosciences) and re-

suspended in RPMI. Monocytes were purified by adhesion for 1 hour followed by washing 6 times in HBSS to remove non-adherent cells. Monocytes were matured for 4 days in 100ng/ml M-CSF and then rested for 1 day in RPMI containing no M-CSF. The resultant monocyte-derived macrophages (MDMs) were infected with *M.tuberculosis* H37RV at an MOI of 1. MMP gene expression was analyzed at 24 hours and MMP secretion measured at 3 days after infection. Conditioned media from *M.tb* infected monocytes (CoMTb) was produced by infecting monocytes at a multiplicity of infection (MOI) of 1. After 24 hours, cell culture medium was harvested, centrifuged at 13,000rpm for 5 minutes and filtered to remove infectious material using a 0.2µm Anopore filter (Whatman, UK) [4].

***Epithelial cell culture***

Normal human bronchial epithelial cells (NHBE) (Lonza, Basel, Switzerland) and the human type II alveolar A549 cell line were acquired as cryopreserved cells and regenerated according to the manufacturers' instructions. A549 cells were maintained in RMPI 1640 with 10% FCS, 2mM glutamine, and 10mg/ml ampicillin. Experiments were performed on cells between passage 8-16 and unless otherwise stated, cell culture supernatants were harvested at 72 hours and MMPs analysed by ELISA or Luminex array. Cell viability was assessed by trypan blue exclusion. Chemical inhibition experiments were performed using dimethyloxalylglycine (DMOG conc. range 0.05-0.5mM) (Inochem, Carnforth, UK), helenalin SC-514 (conc. range 1-100µM) and 3-(2-(4-Adamantan-1-yl-phenoxy)-acetylamino)-4-hydroxybenzoic acid methyl ester, LW6 (conc. range 50-100µM) (all MerckMillipore, Darmstatd, Germany). All inhibitors were dissolved in DMSO according to manufacturers instructions.

### *Hypoxia workstation*

A custom designed hypoxia workstation was commissioned for the biological safety level 3 facility (Coy Laboratories, USA). The workstation comprised a sealed environment with an inbuilt humidified incubator accessed by air-tight gloves. Materials and reagents were passed in and out of the workstation via a sealed airlock, such that the atmosphere remained constant at all times. Temperature and $CO_2$ were maintained at $37^0$C and 5% respectively and $O_2$ levels (by $N_2$ displacement) adjusted according to the experimental requirements. All parameters were monitored by digital sensors throughout and adjustments made automatically. In experiments comparing multiple oxygen levels, a hypoxic incubator was also used for 5% $O_2$ (Galaxy 14S, New Brunswick, UK)

### *Casein Zymography*

To analyze MMP-1 activity, samples were electrophoresed on 0.05% casein gels (Invitrogen) and incubated in collagenase buffer at 37°C for 48h. All gels were run with a 5ng recombinant MMP-1 (Calbiochem, Merck Biosciences, UK) to standardise between gels. Caseinolytic activity was revealed by Coomassie blue staining (Pharmacia) for one hour followed by destain in methanol: acetic acid: water [5].

### *Real-time PCR*

Macrophages were lysed using Tri-Reagent (Sigma-Aldrich, Dorset, UK), and total RNA was extracted using PureLink RNA® Mini Kit (Invitrogen, Paisley, UK). 1µg RNA was reverse transcribed using QuantiTect Reverse Transcriptase Kit (Qiagen, Manchester, UK). qPCR reactions were performed in an ABI Prism 7700 (Applied Biosystems, Paisley, UK). MMP-1

cycle thresholds were quantified by comparison to an MMP-1 standard curve generated using known MMP-1 concentrations generated from a plasmid standard and then standardised to 18S rRNA.  HIF-1α mRNA samples were analysed using the comparative threshold method. The Ct values of both calibrator and samples were normalized to 18S rRNA.

***Measurement of MMP and TIMP concentrations***

Total MMP and TIMP secretion in cell culture supernatants was measured by ELISA (R&D Systems, Abdingdon, UK) or on the Luminex 200 platform using MMP Luminex multiplex array (R&D Systems, Abingdon, UK) according to the manufacturer's instructions.  The minimum level of detection for MMP-1 was 10 pg/ml.

***HIF-1α Western analysis***

Cells were washed 3 times in sterile PBS and then lysed in SDS sample buffer (62.5mM Tris, 2% SDS, 10% glycerol, 50mM DDT, 0.01% bromophenol blue).  For macrophages directly infected with *M.tb* H37RV, samples were sterile filtered through a 0.2μm Anopore filter (Whatman, UK). After heat-denaturation, samples were separated by electrical gradient on a NuPAGE Bis-Tris 4-12% gel (Invitrogen, Paisley, UK), electro-transferred to a nitrocellulose membrane (Amersham, UK) and blocked with 5% milk protein/0.1% Tween-20. The membrane was probed with anti-HIF1α Ab (1:1,000 dilution) (BD Biosiences, UK) overnight, washed, and incubated with goat anti-mouse IgG horseradish peroxidase (HRP)-conjugate secondary Ab (1:1,000 dilution, Jackson ImmunoResearch). Loading control was performed

using the rabbit-anti β-actin Ab (1:5,000 dilution) and goat anti-mouse IgG horseradish peroxidase (HRP)-conjugate secondary Ab (1:5,000 dilution, Jackson ImmunoResearch). Luminescence was detected with the ECL system (Amersham, UK) according to manufacturer's protocol.

***Confocal microscopy***

Primary human monocytes were matured to macrophages in 4-well Permanox plastic chamber slides (Thermo Fisher Scientific Inc. UK). Cells were fixed for 3 days following infection with 4% paraformaldehyde in PBS for 30 minutes. Cells were permeabilized with 0.5% saponin and blocked with 1% BSA for 1 hr. Cells were incubated with anti-MMP-1 primary Ab (1:200, Abcam, UK) overnight, washed and incubated with goat anti-mouse secondary Ab (1:100, Abcam, UK) and labelled with FITC for 2 hrs. Nuclei were visualized using DAPI. The images were scanned under an x60 oil immersion objective and to avoid bleed-through effects, each dye was scanned independently by a Leica TCS SP5 equipped with an argon-krypton mixed gas laser. Image analysis was with Leica confocal software (Leica Microsystems, Germany) and Image J v1.48a.

***Promoter-reporter assay***

A549 cells were transfected with either wild-type, truncation or deletion constructs of the MMP-1 promoter DNA inserted into the firefly luciferase expression plasmid pGL2-basic (gift from Dr. I. Clarke, University of East Anglia, U.K.) and control reporter plasmid, pRL-TK, constitutively expressing *Renilla* luciferase activity, with FuGene 6 (Roche) [6]. Cells were

stimulated with CoMTb in the presence or absence of hypoxia then lysed after 24 h. Luciferase activity was measured using the Dual-Luciferase Reporter Assay System (Promega, Southampton, UK) analyzed with the Bio-Orbit 1253 luminometer (Labtech International). *Renilla* luciferase activity was used to normalize firefly activity to control for transfection efficiency.

***siRNA***

Monocytes were isolated and differentiated into MDMs. MDMs were transfected using DharmaFECT 3 transfection reagent with either non-targeting control siRNA or HIF-1α siRNA smartpool (Dharmacon, FisherScientific, Loughborough, UK), according to the supplier's instructions. Transfection medium was replaced with RPMI at 8hrs and cells allowed to rest for 16hrs, at which time the MDMs were infected with *M.tb* H37Rv (MOI= 1). Cell culture supernatant and cell lysates were harvested for HIF-1α and MMP-1 mRNA and protein secretion analyses. To access transfection efficiency, MDMs were also transfected with 25nM FAM conjugated non-targeting control siRNA, detached from the plates with cell dissociation buffer (Gibco, Invitrogen) and analysed by flow cytometry (FACSCalibur, BD Biosciences).

***Immunohistochemistry***

HIF-1α immunohistochemistry was performed on formalin-fixed, paraffin-embedded lung biopsies from six patients with culture-proved *M.tb* infection and six non-infected control samples. Immunohistochemistry staining was performed using Bond III fully automated staining system with the Bond Polymer Refine Detection system and associated reagents

(Leica Microsystems, Newcastle-Upon-Tyne, UK). Antigen retrieval was carried out using Epitope retrieval pH 6 (ER1) at 100°C for 30mins. The antibody HIF1-α (Abcam AB1) was applied for 30mins at a dilution of 1/500. The detection system uses DAB as chromogen and section were counterstained with haematoxylin.

***Statistics***

Statistical analysis was performed using GraphPad PRISM 6. Data was analyzed with one-way ANOVA and Tukey's post-test performed to account for multiple comparison of variables. A *P* value of 0.05 was considered significant. For all experiments, bars represent mean values of 3 samples ± SD. All graphs are representative of at least duplicate experiments.

## References


1. Patlak, C.S., R.G. Blasberg, and J.D. Fenstermacher, *Graphical Evaluation of Blood-to-Brain Transfer Constants from Multiple-Time Uptake Data.* J Cereb Blood Flow Metab, 1983. **3**(1): p. 1-7.
2. Hudson, H.M. and R.S. Larkin, *Accelerated image reconstruction using ordered subsets of projection data.* Medical Imaging, IEEE Transactions on, 1994. **13**(4): p. 601-609.
3. Avants, B.B., et al., *Symmetric diffeomorphic image registration with cross-correlation: Evaluating automated labeling of elderly and neurodegenerative brain.* Medical Image Analysis, 2008. **12**(1): p. 26-41.
4. Elkington, P., *Filter sterilization of highly infectious samples to prevent false negative analysis of matrix metalloproteinase activity.* J Immunol Methods, 2006. **Feb 20**: p. 309(1-2).
5. Troeberg, L. and H. Nagase, *Zymography of Metalloproteinases*, in *Current Protocols in Protein Science*. 2001, John Wiley & Sons, Inc.
6. O'Kane, C.M., et al., *STAT3, p38 MAPK, and NF-kappaB drive unopposed monocyte-dependent fibroblast MMP-1 secretion in tuberculosis.* Am J Respir Cell Mol Biol, 2010. **43**(4): p. 465-74.

| | Patient 1 1P | Patient 2 | Patient 3 | Patient 4 | Patient 5 |
|---|---|---|---|---|---|
| **Sex (M/F)** | M | F | F | F | F |
| **Age (years)** | 49 | 22 | 31 | 37 | 62 |
| **Country of origin** | Romania | Mongolia | Nepal | Philippines | Morocco |
| **Height (cm)** | 178 | 160 | 153 | 157 | 156 |
| **Weight (kg)** | 64 | 53.2 | 44.9 | 45.3 | 48 |
| **Duration of symptoms** | 6 months | 3 months | 1 month | 1 month | 1 month |
| **Symptoms** | | | | | |
| **Cough** | + | + | + | + | + |
| **Weight loss** | + | - | + | + | - |
| **Fever** | + | + | + | + | + |
| **Haemoptysis** | + | - | - | - | - |
| **GI symptoms** | - | - | + | - | - |
| **Lethargy** | - | - | - | - | + |
| **Smoking status** | Current 30 pack years | Never | Never | Never | Never |
| **CXR** | | | | | |
| **Consolidation** | + | + | + | + | + |
| **Cavitation** | + | + | - | + | + |
| **Fibrosis** | - | - | + | - | - |
| **Sputum smear** | AFB +++ | AFB +++ | AFB + | AFB + | AFB +++ |
| **Days to *M.tb* culture positivity** | 3 days | 7 days | 26 days | 7 days | 5 days |
| **Antibiotic sensitivity** | Fully sensitive | Resistant isoniazid + streptomycin | Fully sensitive | Resistant rifampicin, isoniazid + prothionomide | Fully sensitive |

**Supplementary Table 1.** Patient demographics, chest radiograph and microbiology results

| | Patient 1t 1 | Patient 2 | Patient 3 | Patient 4 | Patient 5 |
|---|---|---|---|---|---|
| **Haematology** | | | | | |
| **Hb (g/dl)** | 10.8 | 10.2 | 10.1 | 12.7 | 10.4 |
| **WCC (x$10^9$g/l)** | 7.5 | 7.7 | 10.4 | 16.4 | 10.7 |
| **Neutrophil (x$10^9$g/l)** | 6.0 | 4.4 | 7.3 | 12.8 | 8.7 |
| **Biochemistry** | | | | | |
| **Na (mmol/l)** | 129 | 141 | 134 | 132 | 132 |
| **K+ (mmol/l)** | 4.0 | 4.1 | 4.8 | 4.0 | 4.0 |
| **Urea (mmol/l)** | 3.5 | 3.5 | 2.4 | 3.1 | 8.1 |
| **Cr (µmol/l)** | 52 | 53 | 55 | 65 | 97 |
| **CRP (mg/l)** | 189 | 10.6 | 150 | 69 | 312 |
| **Liver function** | | | | | |
| **Bilirubin (µmol/l)** | 10 | n/a | 6 | 11 | 12 |
| **ALT (IU/l)** | 39 | n/a | 13 | 29 | <6 |
| **ALP (IU/l)** | 135 | n/a | 95 | 114 | 113 |
| **Total protein (g/l)** | 62 | n/a | 70 | 72 | 75 |
| **Albumin (g/l)** | 39 | n/a | 27 | 24 | 49 |
| **HIV status** | Negative | Negative | Negative | Negative | Negative |
| **Vitamin D (nmol/l)** | 26.0 | 28.6 | 24.3 | 25.9 | 14.9 |

**Supplementary Table 2.** Blood analysis at time of presentation n/a, not available

**Supplementary Table 3.** Appearance on CT and binary hypoxic scale for each lesion examined.

| **Patient number** | **Lesion number** | **CT appearance** | **Hypoxic*** |
|---|---|---|---|
| 1 | 1 | Consolidation | - |
| | 2 | Consolidation | + |
| | 3 | Consolidation | - |
| | 4 | Consolidation | - |
| 2 | 1 | Consolidation | + |
| | 2 | Consolidation | + |
| | 3 | Cavity wall | + |
| | 4 | Consolidation | + |
| 3 | 1 | Consolidation | + |
| | 2 | Consolidation | + |
| | 3 | Consolidation | + |
| | 4 | Consolidation | + |
| | 5 | Consolidation | - |
| | 6 | Consolidation | - |
| | 7 | Consolidation | + |
| 4 | 1 | Cavity wall | + |
| | 2 | Cavity wall | + |
| 5 | 1 | Cavity wall | - |
| | 2 | Cavity wall | - |
| | 3 | Consolidation | + |
| | 4 | Consolidation | + |
| | 5 | Consolidation | - |

*The binary hypoxic scale denotes whether the lesion ROI Ki value is above a hypoxia threshold (0.00037 $min^{-1}$) determined from the mean + 3SD of the Ki values within the lateral muscle background ROIs, assumed to represent normoxic tissue.

## Figure Legends Supplementary figures

**Figure S1. Hypoxia does not cause cytotoxicity in NHBE cells**

NHBE cells were stimulated with CoMTb and incubated in normoxia or hypoxia (1% or 5% pO2; 5% CO2). Supernatant were harvested at 72 hrs. Hypoxia did not cause an increase in LDH release in either control or CoMTb stimulated cells.

**Figure S2. DMOG up-regulates MMP-9 secretion in NHBE cells stimulated with CoMTb.**

NHBE cells were pre-incubated with DMOG (0.25mM) for 2 hours before stimulation with CoMTb. Supernatants were harvested at 72 hours. (A) Gelatin zymography shows DMOG increases MMP-9 gelatinolytic activity compared to control cells. A marked increase in MMP-9 proteolytic activity is seen in CoMTb stimulated cells. (B) DMOG and CoMTb stimulation increases MMP-9 secretion compared to cells treated with DMOG or CoMTb alone. *$p<0.05$

**Figure S3. Hypoxia and DMOG up-regulates MMP-1 secretion in A549 cells stimulated with CoMTb.**

A549 cells were incubated in hypoxia ($pO_2$ 1%) or pre-incubated with DMOG (0.25mM) for 2 hours and then stimulated with CoMTb. Supernatants were harvested at 72 hours and MMP-1 concentrations measured by ELISA. (A) Hypoxia significantly increases MMP-1 secretions compared to cells incubated in normoxia. (B) DMOG significantly up-regulates MMP-1 secretion compared to cells treated with DMOG or CoMTb alone. ***$p<0.001$

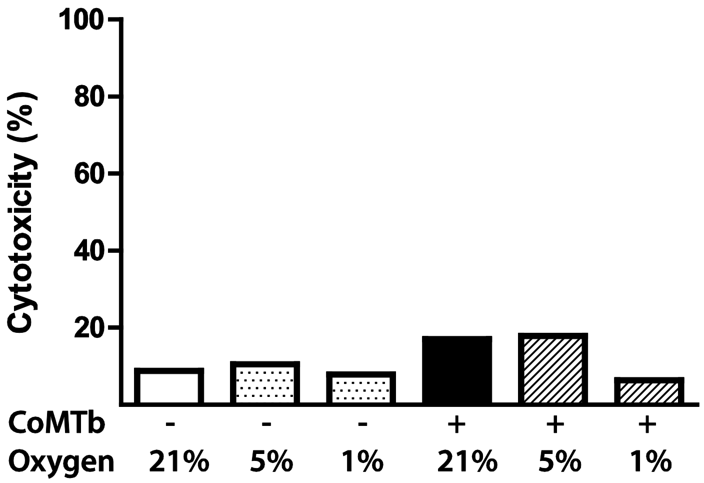


Supplementary Figure 1

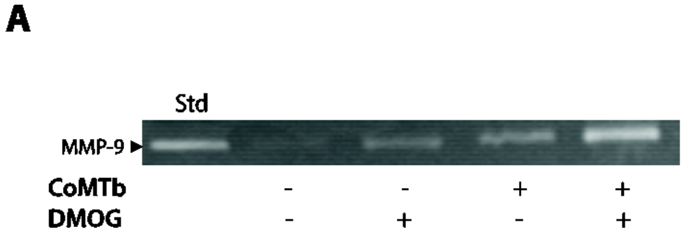


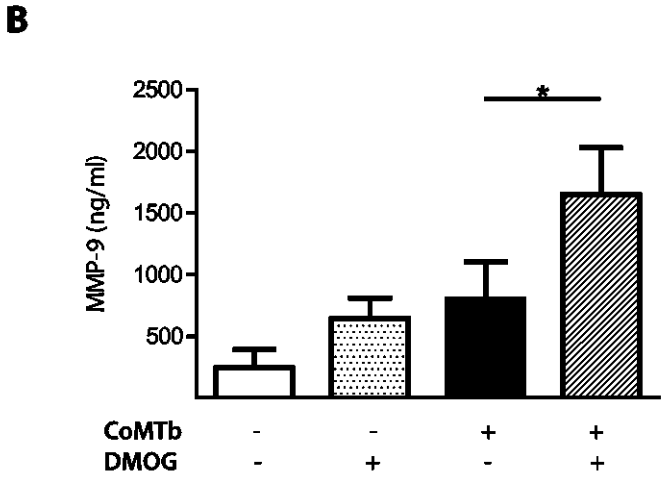


Supplementary figure 2

**A.**

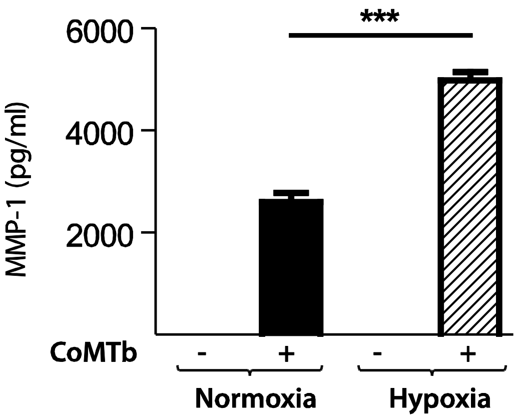


**B.**

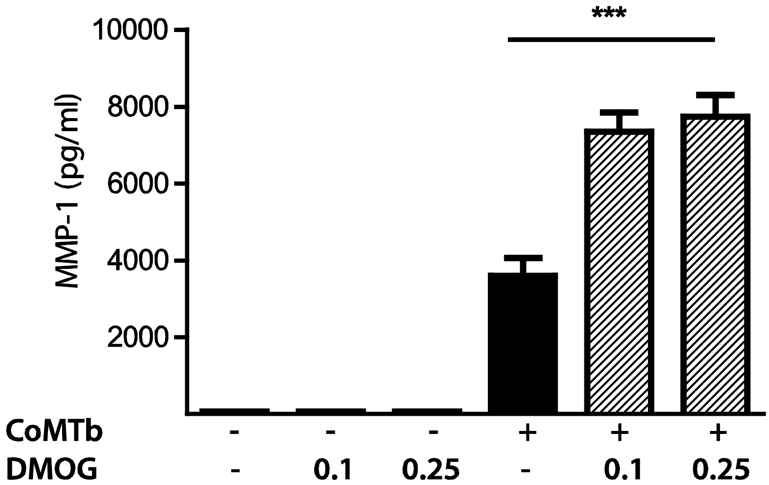


Supplementary figure 3
